# Long lasting clinical response to chemotherapy for advanced uterine leiomyosarcoma: a case report

**DOI:** 10.1186/1752-1947-7-29

**Published:** 2013-01-24

**Authors:** Claudio Ridolfi, Giuseppe Pasini, Fabrizio Drudi, Eleonora Barzotti, Carlotta Santelmo, Antonio Polselli, Alberto Ravaioli

**Affiliations:** 1 Oncology Department, ‘Infermi’ Hospital, Via Settembrini 2, Rimini, 47923, Italy; 2 Oncology Department, ‘Cervesi’ Hospital, Via Ludwig Van Beethoven 1, Cattolica, RN 47841, Italy; 3 IRST (The Cancer Institute of Romagna), Via Piero Maroncelli 40, Meldola, FC 47014, Italy

**Keywords:** Advanced leiomyosarcoma, Chemotherapy, Response

## Abstract

**Introduction:**

Uterine leiomyosarcoma is one of the most frequent uterine sarcomas. In the metastatic setting it is sensitive to doxorubicin, ifosfamide, gemcitabine, docetaxel and a few other drugs, but time to progression is generally short. For this reason prognosis is often poor and there are few reports in the literature of long responders.

**Case presentation:**

We report a case of a 40-year-old Caucasian woman with metastatic uterine leiomyosarcoma who began treatment six years before the presentation of this case report and for the following six years underwent ten lines of chemotherapy, achieving excellent results and a good quality of life. Among the treatments administered we observed a long response to temolozomide, an unconventional drug for this kind of disease.

**Conclusion:**

Although there are few chemotherapeutic options for the management of metastatic uterine leiomyosarcoma, a small number of patients have an unexpected long lasting response to treatment. For this reason further research is needed to identify new therapeutic agents and the predictive factors for the achievement of response.

## Introduction

Uterine sarcomas are rare tumors with a poor prognosis; they represent less than 3% of all female genital tract malignancies and only 8.4% of uterine cancers [1,2]. They can be classified into two groups: smooth muscle tumors and endometrial stromal tumors. The first group includes benign leiomyomas, smooth muscle tumors of uncertain malignant potential, and leiomyosarcomas (LMS). The second group is composed of benign endometrial stromal nodules, endometrial stromal sarcomas and undifferentiated endometrial sarcomas [1].

Treatment for early-stage disease is surgery, with total abdominal hysterectomy with or without bilateral salpingo-oophorectomy. The role of adjuvant chemotherapy or local radiotherapy is still controversial [1-3].

In the metastatic setting, treatment of a uterine sarcoma includes resection of metastases, chemotherapy, hormone therapy and targeted therapy [1,3,4]. Among the chemotherapies used, the drugs that have shown the best results are doxorubicin with or without ifosfamide, gemcitabine and docetaxel and, more recently, trabectedin and pazopanib [1,4-7]. Although literature data underline the possibility of a long lasting response to different types of therapy using drug associations, few papers report encouraging results beyond second-line chemotherapy.

We describe a case of a long-term responder to a wide variety of drugs, highlighting that it is possible to offer more than two lines of chemotherapy to some patients.

## Case presentation

A 40-year-old premenopausal Caucasian woman without comorbidities underwent surgical treatment seven years before the presentation of this case report for a clinical diagnosis of uterine myoma. The postoperative histological examination revealed the presence of a LMS and a hysterectomy with lymphadenectomy was thus performed. The tumor was estrogen and progesterone receptor positive. A staging computed tomography scan detected bilateral, multiple lung metastases of about four to six mm. Six years before the presentation of this case report the patient underwent first-line chemotherapy with Adriamycin® (doxorubicin) and ifosfamide but after six cycles of therapy the lung metastases had increased in size and a new lesion had appeared in the abdomen near the right psoas muscle. It was decided to perform a partial resection of the lung metastases (with histological confirmation) and an ovariectomy.

Second-line treatment with sorafenib was begun but was rapidly discontinued because of toxicity and treatment with dacarbazine was started as third-line. After three cycles, progression in the lung metastases was detected and continuous infusion of ifosfamide was administered as fourth-line therapy for the following two months. Then the patient was discharged from a National Oncologic Centre to be treated with palliative care.

When the patient arrived in our Department, further progression in the abdomen and chest caused an inability to walk due to nerve impingement and pain and a fifth-line of treatment was begun with docetaxel and gemcitabine. In these 11 months the patient showed a partial response and regained the ability to walk. Disease stabilization in the following six months was achieved with anastrozole (sixth line) and at the next progression the patient was treated with paclitaxel and liposomal doxorubicin (seventh line) for 19 cycles and then with trabectedin (eighth line) for eight cycles, until May of two years before the presentation of this case report. It is noteworthy that more than four years had passed since the initial diagnosis and the patient continued to feel well (with only right leg pain, and three points on visual analogue scale, VAS). After eight cycles of trabectedin the lung metastases and pelvic lesion increased to three cm and 18cm, respectively, causing bilateral ureteral compression and an increase in leg pain (VAS eight to nine).

**Figure 1:**
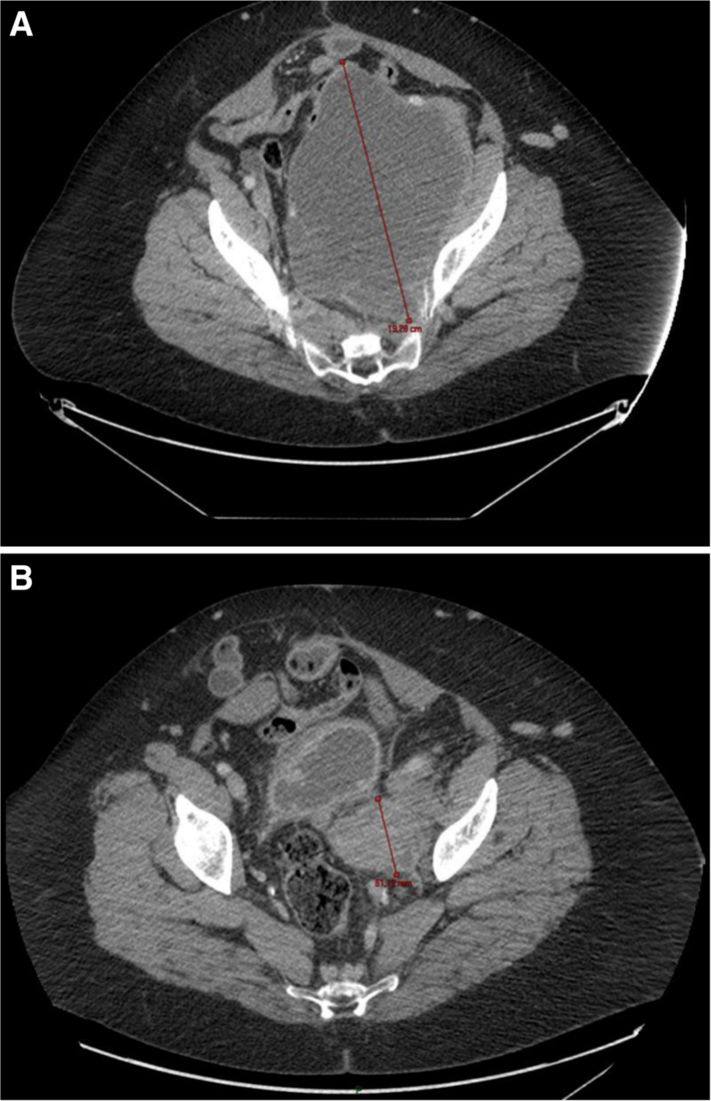
**Effect of temozolomide on pelvic metastasis. A**: before the beginning of treatment. **B**: after 11 months of treatment.

**Figure 2:**
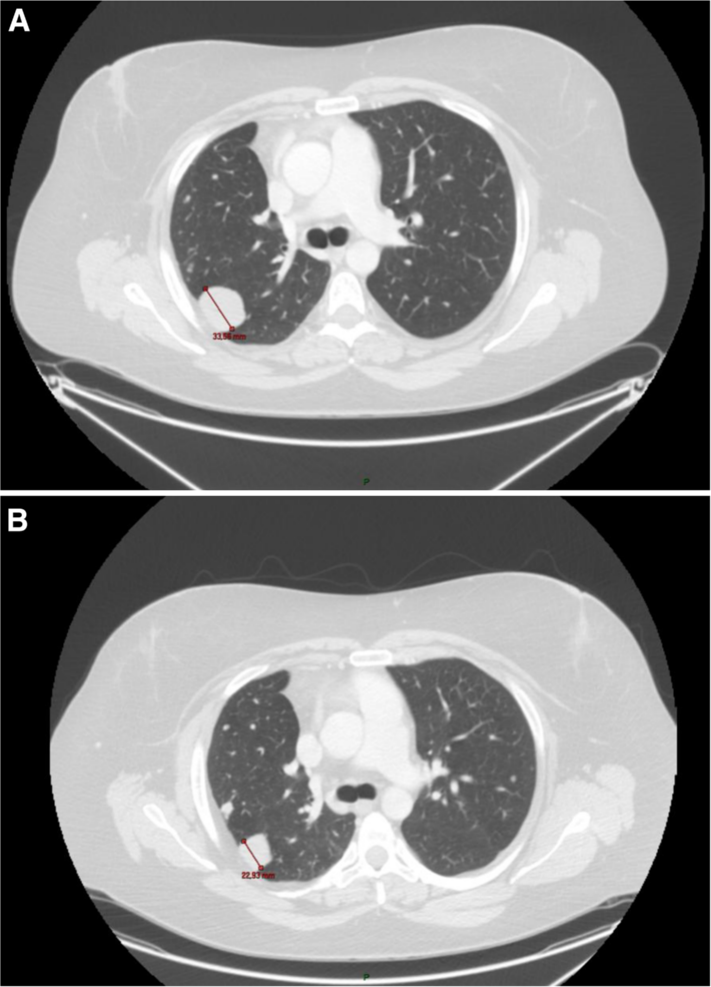
**Effect of temozolomide on one of the lung metastases. A**: before the beginning of treatment. **B**: after 11 months of treatment.

In July of two years before the presentation of this case report the patient’s performance status (PS) was two on the Eastern Cooperative Oncology Group (ECOG) scale and a bilateral nephrostomy was performed. A ninth-line of treatment was started with temozolomide at a dose of 150mg/m^2^/day for five days every four weeks. Despite the negative status of O6-methylguanine-deoxyribonucleic acid (DNA)-methyltransferase promoter methylation (this kind of test is performed for patients with glioblastoma multiforme to predict response to temozolomide [8]), a rapid response to therapy was observed with a reduction in the pelvic lesion, resolution of pain and removal of the nephrostomies (Figures 1 and 2). The patient returned to her normal life and was able to use her bicycle and to perform activities without leg pain or fatigue (ECOG PS 0) that would previously have been unthinkable. Treatment with temozolomide was completed after 24 months, with very good tolerance, but further progression was detected. The patient recently began a new line of therapy (the tenth) with pazopanib and is currently doing well, six years after the start of treatment.

## Discussion

Our case underlines the role of conventional and unconventional drugs for the treatment of metastatic LMS. Temozolomide has been tested in numerous clinical trials on metastatic soft tissue sarcomas (STSs).

In 2011, Penel *et al*. [9] reported their analysis of new regimens used in patients with STSs during the past 10 years and temozolomide at a dose of 75 to 100mg/m^2^/day was considered a ‘promising drug’.

The Soft Tissue and Bone Sarcoma Group of the European Organization for Research and Treatment of Cancer [10] evaluated the use of 750mg/m^2^ of oral temozolomide divided over five days every four weeks in 31 patients with advanced STSs. One partial response for eight cycles was observed in a patient with retroperitoneal LMS metastatic to breast, skin, and liver. Nine patients of varying histologies had stable disease, and 21 patients progressed to therapy. Nausea and vomiting were the most commonly reported non-hematologic adverse events; there were no episodes of neutropenic fever or grade three or four hematologic toxicity.

Talbot *et al*. [11] treated 25 patients with STSs with twice daily temozolomide for five days every four weeks. Two patients had partial responses (8%), three patients (12%) experienced stable disease for over six months, and two patients had mixed responses (8%). All of these responding patients had LMS. No treatment-related deaths or grade four toxicity were observed.

In their study Trent *et al*. [12] evaluated patients with gastrointestinal stromal tumors (GIST) and other STSs treated with temozolomide at a dose of 85mg/m^2^/day for 21 days followed by seven days of rest. Among 39 evaluable patients with non-GIST tumors, 18 had metastatic LMS. Two responses (11%) were observed in the other STSs subgroup and stable disease was observed in 33% of patients. One partial response lasting for seven months was seen in a patient with metastatic pelvic LMS to the lung. Grade three or four hematologic toxicity was experienced in 10% of patients. The most common non-hematologic adverse event was fatigue, which was observed in 14% of patients.

Garcia del Muro *et al*. [13] evaluated oral temozolomide given at 75mg/m^2^/day for six weeks in 43 patients with STSs and 18 patients with GIST. In the first group, seven patients obtained a partial response, eight patients had stable disease and 28 patients had progressive disease. Responses were seen in five of the 11 patients who had gynecologic LMS. The median duration of response was 12.5 months. Severe hematological toxicities for the 61 patients of two groups were grade three or four granulocytopenia or thrombocytopenia (nine patients), grade four lymphocytopenia (24 patients) and grade three or four anemia (seven patients). Non-hematologic toxicity was represented by nausea and vomiting (34 patients) and fatigue (35 patients). No toxic deaths or episodes of febrile neutropenia occurred.

Anderson and Aghajanian [14] evaluated patients with advanced LMS treated with temozolomide at a dose of 50 to 75mg/m^2^/day for six weeks followed by a two-week rest or temozolomide at a dose of 150 to 300mg/m^2^/day for five days every four weeks. Among 12 patients treated with continuous low-dose temozolomide, one patient had a partial response and the duration was four months, four patients demonstrated stable disease and seven patients progressed while on therapy. Among seven patients treated with bolus-dose temozolomide, one patient had a near complete response, four patients experienced stable disease and two patients progressed while on therapy. There were no toxicity-associated admissions, deaths, neutropenia or neutropenic fever for either dosing schedule.

On the basis of these works we decided to start the treatment with temozolomide and in our patient it obtained the best response and gave a good quality of life without significant toxicity, indicating that its use could be considered after failure of standard treatment options.

## Conclusion

This case report highlights the fact that some patients with uterine LMS can have an exceptionally good response to chemotherapy. On the basis of our experience temozolomide should be used in these patients.

## Consent

Written informed consent was obtained from the patient for publication of this case report and accompanying images. A copy of the written consent is available for review by the Editor-in-Chief of this journal.

## Abbreviations

ECOG: Eastern Cooperative Oncology Group; GIST: Gastrointestinal stromal tumors; LMS: Leiomyosarcoma; PS: Performance status; STSs: Soft tissue sarcomas; VAS: Visual analogue scale.

## Competing interests

The authors declare that they have no competing interests.

## Authors’ contributions

GP, CS, AP and AR took care of the patient. AR and CR described and wrote the case report. FD and EB revised the literature. AR and CR performed the last revision. All authors have read and approved the final manuscript.
